# Molecular Mechanisms for Vascular Development and Secondary Cell Wall Formation

**DOI:** 10.3389/fpls.2016.00356

**Published:** 2016-03-22

**Authors:** Jung Hyun Yang, Huanzhong Wang

**Affiliations:** ^1^Department of Plant Science and Landscape Architecture, University of ConnecticutStorrs, CT, USA; ^2^Institute for Systems Genomics, University of ConnecticutStorrs, CT, USA

**Keywords:** vascular, secondary cell wall, development, transcriptional regulation, *Arabidopsis*

## Abstract

Vascular tissues are important for transporting water and nutrients throughout the plant and as physical support of upright growth. The primary constituents of vascular tissues, xylem, and phloem, are derived from the meristematic vascular procambium and cambium. Xylem cells develop secondary cell walls (SCWs) that form the largest part of plant lignocellulosic biomass that serve as a renewable feedstock for biofuel production. For the last decade, research on vascular development and SCW biosynthesis has seen rapid progress due to the importance of these processes to plant biology and to the biofuel industry. Plant hormones, transcriptional regulators and peptide signaling regulate procambium/cambium proliferation, vascular patterning, and xylem differentiation. Transcriptional regulatory pathways play a pivot role in SCW biosynthesis. Although most of these discoveries are derived from research in *Arabidopsis*, many genes have shown conserved functions in biofuel feedstock species. Here, we review the recent advances in our understanding of vascular development and SCW formation and discuss potential biotechnological uses.

## Introduction

Plant vascular tissues are composed of xylem, phloem and the intervening procambial or cambial cells (Eames and MacDaniels, 1947). The proliferation of stem cells in the vascular meristem produces progeny cells, which either maintain their stem cell property or differentiate into xylem toward the center and phloem toward the periphery of plant stems (Elo et al., 2009; Miyashima et al., 2013; Jouannet et al., 2015). During the differentiation process, xylem fibers and tracheary elements (TEs), including vessels and tracheids, develop secondary cell walls (SCW; Ohashi-Ito and Fukuda, 2014). The development of TEs and xylary fibers undergoes a programed cell death (PCD) process (Schuetz et al., 2013). Compared to the thin primary cell walls, the SCW is much thicker and accounts for the majority of cellulosic biomass that serves as a renewable resource for biofuel production (Demura and Ye, 2010; Carpita, 2012).

Our understanding of vascular development including hormonal response, peptide signaling, and transcriptional regulation has advanced significantly since the publication of a few recent reviews (Kondo et al., 2014b; Ruzicka et al., 2015). Rapid progress have also been made in the genetic regulation of SCW biosynthesis due to the growing interest in clean bioenergy and biofuels (Somerville, 2007; Carroll and Somerville, 2009; Pauly and Keegstra, 2010). In fact, vascular development and SCW formation are closely related biological processes that can be regulated by the same signaling pathway (Ito et al., 2006; Etchells and Turner, 2010). In this mini-review, we focus on progress in elucidating the regulatory pathways involved in vascular development, xylem differentiation and SCW deposition.

## The Initiation of Vascular Procambium

Plant stems contain most of the collectable terrestrial biomass, but the study of vascular procambium initiation in the stem is impeded because these cells are imbedded under layers of other tissues and are difficult to access. Most of the current knowledge on procambium initiation and regulation is derived from studies in embryos, root apical meristems, and leaf venation systems. Some of the genes and signaling pathways, such as the Class III homeodomain leucine zipper (HD-ZIP III) and the CLAVATA 3 (CLV3)/EMBRYO SURROUNDING REGION (ESR) related (CLE) signaling pathway, function in multiple tissues, and therefore appear to be more broadly involved in probambium development in general (Zhang et al., 2014; Ruzicka et al., 2015). The vascular procambium develops during embryogenesis and determines vascular patterning in postembryonic growth. In early globular embryos, division of the four inner cells generates procambial/provascular initials (Hardtke and Berleth, 1998; Berleth et al., 2000; Jouannet et al., 2015). These initial cells further divide periclinally, increase in number and form the first vascular strands in a pattern similar to what is later observed in young seedlings. During postembryonic development, the initiation of procambial strands in leaf primordia and root meristems are extensively studied and reviewed elsewhere (Cano-Delgado et al., 2010; Kondo et al., 2014b; Jouannet et al., 2015; Ruzicka et al., 2015).

**FIGURE 1:**
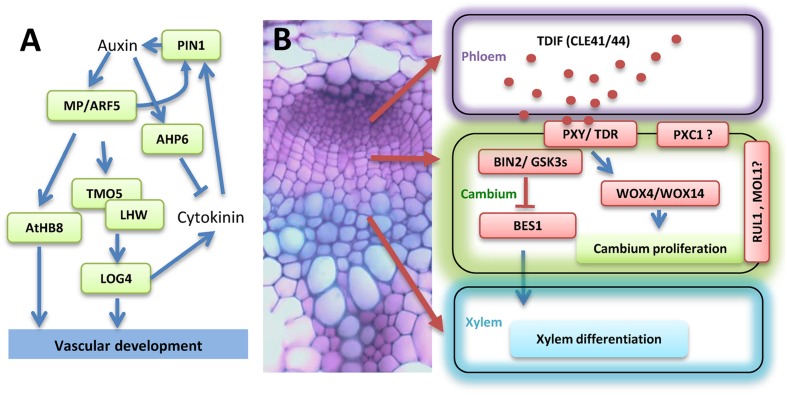
**Illustration of the transcriptional and hormonal regulation of vascular development. (A)** Hormonal and transcriptional control of vascular procambium initiation. **(B)** Regulation of vascular procambium proliferation, xylem differentiation, and vascular patterning by peptide TDIF (red dots in the phloem) signaling. A cross section of the *Arabidopsis* stem to demonstrate cell types is shown on the left. The localization of different components of the TDIF signaling pathways is illustrated on the right. Abbreviations for gene names are specified in the text.

The plant hormone auxin, mainly indole acetic acid (IAA), regulates the initiation of vascular procambial cells. Mutation of the auxin responsive transcription factor (TF) *AUXIN RESPONSE FACTOR 5* (*ARF5*)/*monopteros* (*MP*) inhibits vascular procambial cell formation in embryos (Hardtke and Berleth, 1998) (**Figure 1A**). The expression of *ARF5/MP* is restricted to the provascular initials (Hamann et al., 2002). Furthermore, the expression of *ARF5/MP* is upregulated in the developing procambium cells and is preceded by auxin accumulation (Hardtke and Berleth, 1998). ARF5/MP binds to the promoter of *ARABIDOPSIS THALINA HOMEOBOX 8* (*ATHB8*) and directly regulate its expression through an auxin responsible element (ARE, TGTCTG; Donner et al., 2009). In addition to auxin signaling, auxin transport is also important to procambium development. During embryogenesis, the auxin eﬄux carrier PIN-FORMED1 (PIN1) protein is polarly localized in the inner cells of the pre-procambium (Friml, 2003). The expression level of *PIN1* is dramatically reduced in *mp* mutant plants (Wenzel et al., 2007), suggesting that MP may regulate *PIN1* at the transcriptional level (**Figure 1A**). TARGET OF MONOPTEROS 5 (TMO5), a basic helix-loop-helix (bHLH) TF, is identified as a direct target of ARF5/MP. TMO5 is expressed in procambium initials in globular stage embryos, and is restricted to the xylem precursor cells in the postembryonic root (Schlereth et al., 2010). TMO5 physically interacts with another bHLH TF LONESOME HIGHWAY (LHW) to control the periclinal divisions (De Rybel et al., 2013; Ohashi-Ito et al., 2013). Ectopic expression of TMO5 and LHW causes periclinal cell divisions in other tissues, indicating conserved functions of the TMO5/LHW dimer (De Rybel et al., 2013).

Cytokinin (CK) is another major plant hormone that is critical to procambium initiation. The direct downstream target of TMO5/LHW dimer was identified as LOG4 (**Figure 1A**), a rate-limiting enzyme in CK biosynthesis (De Rybel et al., 2014; Ohashi-Ito et al., 2014). In the *Arabidopsis* root procambium, CK promotes the bisymmetric distribution of PIN1 and PIN7, and as a result, channels auxin toward the axis of xylem precursor cells. In contrast, auxin positively regulates the expression of an inhibitor of CK signaling, *AHP6* (**Figure 1**). This mutually inhibitory feedback loop between auxin and CK sets distinct boundaries and defines the organization of the root vascular cylinder (Bishopp et al., 2011). In addition to auxin and CK, other hormones may also play a role in procambium initiation (Jouannet et al., 2015; Ruzicka et al., 2015). The aforementioned hormonal regulations were derived from studies in embryos and roots. It would be interesting to investigate how perturbation of these pathways affect procambium and cambium development in stems.

## The Development of Vascular Tissues in the *Arabidopsis* Stem

### The Development and Patterning of the Vascular Bundle

Vascular bundles of the *Arabidopsis* stem are organized in a collateral pattern with the procambium located between xylem and phloem tissues. During secondary growth, vascular cambia develop in both fascicular (vascular bundles) and interfascicular regions and form a continuous ring, during which process auxin plays a critical role (Mazur et al., 2014). Class III homeodomain leucine zipper (HD-ZIP III) genes, i.e., *ATHB8, PHABULOSA (PHB), PHAVOLUTA (PHV), REVOLUTA (REV)*, and *ATHB15*, regulate vascular tissue development and adaxial-abaxial patterning in *Arabidopsis*. These genes are shown to be induced by auxin (Donner et al., 2009; Ursache et al., 2014). These HD-ZIP III TFs promote adaxialization and cause the formation of amphivasal bundles (phloem surrounded by xylem) in gain of function mutants (McConnell et al., 2001). In contrast, amphicribral vasculature (xylem surrounded by phloem) were observed in loss of function mutants of HD-ZIP III genes, such as in the triple mutant of *phb phv rev* (Emery et al., 2003). The function of AtHB15 may be different from its family members, especially REV. The triple mutant *phb phv athb15* develops amphivasal vasculature that is opposite to the *phb phv rev* (Green et al., 2005; Prigge et al., 2005). The expression of *HD-ZIP III* TFs is regulated by micro-RNA 165/166 (*miR165/166*). Activation tagging of *miR165b*, *miR166a*, and *miR166g* promote the cleavage of the transcripts of *PHB*, *PHV*, and *AtHB15* resulting in internalized amphivasal bundles (Kim et al., 2005; Williams et al., 2005; Du et al., 2015). The transcripts of *REV* and *ATHB8* are less affected by activation tagging of miR165/166 due to unknown mechanisms (Du and Wang, 2015).

### The Proliferation and Maintenance of Vascular Procambium

The proliferation of vascular procambium and subsequently xylem differentiation is regulated by a CLE peptide signaling. In *Arabidopsis*, *CLE41* and *CLE44* encode the dodeca-peptide TE differentiation inhibition factor (TDIF), which activity was originally identified from a *Zinnia* cell culture system (Ito et al., 2006). TDIF is synthesized in the phloem, diffuses into the cambial tissue, and binds to its receptor, a leucine-rich repeat receptor like kinase (LRR-RLKs) named PHLOEM INTERCALATED WITH XYLEM (PXY; Ito et al., 2006; Fisher and Turner, 2007; Hirakawa et al., 2008). TDIF signaling activates the expression of *WUSCHEL-RELATED HOMEOBOX 4* (*WOX4*) and *WOX14*, resulting in the promotion of cambial cell proliferation (Hirakawa et al., 2010; Etchells et al., 2013) (**Figure 1B**). Mutation of *WOX4* represses procambium proliferation in the hypocotyl of 7-day-old seedlings (Hirakawa et al., 2010; Etchells et al., 2013). However, overexpression of *WOX4* does not significantly increase procambial cell proliferation in *Arabidopsis* hypocotyls (Hirakawa et al., 2010). It is possible that other factors, such as HAIRY MERISTEM (HAM; Zhou et al., 2015), are required for WOX4 function. HAM family TFs act as conserved interacting cofactors with WOX proteins and may be essential for all stem cell niches in plant (Zhou et al., 2015). The TDIF peptide also regulates vascular tissue organization as overexpression of *CLE41* or *CLE44* with a ubiquitous promoter or a xylem specific promoter leads to a loss of cell division orientation (Etchells and Turner, 2010). Three other LRR-RLKs, *PXY-CORRELATED 1* (*PXC1*), MORE LATERAL GROWTH 1 (MOL1) and REDUCED IN LATERAL GROWTH 1 (RUL1) were shown to be involved in regulating cambium activity (Agusti et al., 2011; Wang et al., 2013). Further analysis of these receptor like kinases may help to better understand the maintenance of procambial and cambium cells (**Figure 1B**).

### The Differentiation of Xylem Cells

The mechanism of how TDIF-TDR signaling represses xylem differentiation was revealed recently. BRASSINOSTEROID-INSENSITIVE 2 (BIN2) was identified as an interacting partner of TDR/PXY in a yeast two-hybrid screening (Kondo et al., 2014a). BIN2 is a Glycogen Synthase Kinase 3 (GSK3) protein and is directly associated with TDR/PXY at the plasma membrane. BES1 (BRI-EMS-SUPPRESSOR 1) is one of the BIN2 downstream TFs in the brassinosteroid (BR) signaling pathway (Li and Nam, 2002; Yin et al., 2002), and positively regulates xylem cell differentiation (Kondo et al., 2015). TDIF binding to its receptor TDR/PXY disassociates BIN2 from the complex, suppresses the function of BES1, and subsequently inhibits xylem formation (Kondo et al., 2014a).

## Transcriptional Regulation of SCW Development

**FIGURE 2:**
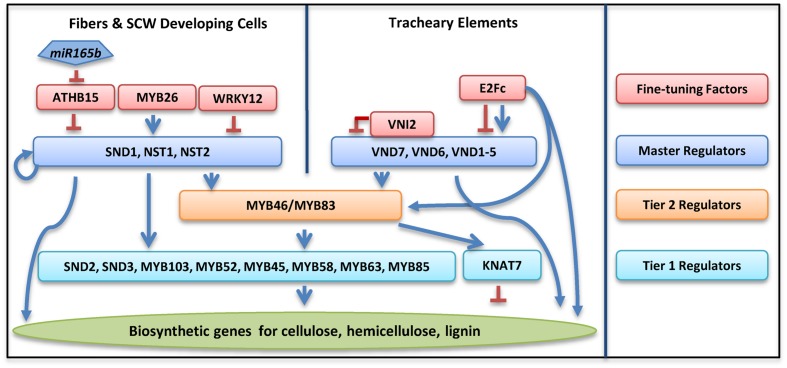
**Transcriptional regulatory networks in regulating secondary cell wall biosynthesis in *Arabidopsis thaliana***. Colored rectangles represent transcription factors in different tiers as specified in the column on the right. Blue arrows denote positive regulation, while a red line with blunt ends denotes negative regulation.

Secondary cell wall deposition is regulated by a large number of TFs through both hierarchical and non-hierarchical regulatory networks (Wang and Dixon, 2012; Zhong and Ye, 2015). At least three layers of regulators, including NAC (NO APICAL MERISTEM, ATAF1, ATAF2, and CUP-SHAPED COTYLEDON 2) domain master regulators in tier 3, two MYB domain regulators in tier 2 and many other regulators in tier 1, are directly involved in regulating SCW biosynthetic genes (**Figure 2**).

### The NAC Domain (Tier 3) Master Regulators

Three NAC domain TFs are defined as master regulators for their function in regulating all three components, i.e., cellulose, hemicellulose, and lignin biosynthesis in xylary fibers (Wang et al., 2011; Wang and Dixon, 2012; Zhong and Ye, 2015). These three NACs are *NAC SECONDARY WALL THICKENING PROMOTING FACTOR 1* (*NST1*), *NST2*, and *NST3/SECONDARY WALL-ASSOCIATED NAC DOMAIN PROTEIN 1* (*SND1*; Zhong et al., 2006, 2007b; Mitsuda et al., 2007). The SCW deposition is disturbed in the vascular and interfascicular fibers of the *nst1nst3* double knockout plants, while ectopic overexpression of *NST1* or *SND1* leads to ectopic SCW formation in a variety of tissues (Zhong et al., 2006, 2007b; Mitsuda et al., 2007). In *Arabidopsis* anther endothecium, secondary wall thickening is controlled by NST1 and NST2 (Mitsuda et al., 2005). In xylem vessels, VASCULAR-RELATED NAC DOMAIN (VND) proteins regulate both SCW biosynthesis and PCD (Yamaguchi et al., 2010a). VND6 and VND7 positively regulate xylem vessel differentiation (Kubo et al., 2005). In *Arabidopsis*, ectopic expression of *VND6* and *VND7* triggers metaxylem and protoxylem formation, respectively (Kubo et al., 2005; Yamaguchi et al., 2010a). VND6 and VND7 activate the expression of a broad range of genes involved in PCD, such as xylem-specific papain-like cysteine peptidase (XCP1; Funk et al., 2002; Yamaguchi et al., 2011). Other VND family members, i.e., VND1 to VND5, function redundantly with VND6 and VND7 in vessel development (Zhou et al., 2014).

### The MYB Domain Second Level (Tier 2) Regulators

MYB46 and MYB83 are the second level regulators downstream of the NAC domain master regulators (Zhong et al., 2007a; Zhong and Ye, 2012). SND1 directly binds to the promoter of MYB46 and activates its expression (Zhong et al., 2007a; Wang et al., 2011). Overexpression of *MYB46* or *MYB83* leads to over-accumulation of all three major SCW components, indicating that both of these MYBs also function as master switches (Zhong et al., 2007a; Zhong and Ye, 2012). These MYBs are also direct targets for VND6 and VND7 (Ohashi-Ito et al., 2010; Yamaguchi et al., 2011), indicating that they are important for SCW formation in both vessels and xylary fibers. Consistent with this observation, simultaneous knockout of *MYB46* and *MYB83* results in a more severe phenotype than those observed from the *nst1nst3* double mutant (Zhong and Ye, 2012).

### Other Regulators (Tier 1) for SCW Biosynthesis

Many other TFs function downstream the NAC and MYB domain master regulators (Zhong et al., 2008; Ko et al., 2009). Among these regulators, *SND2*, *SND3*, and *MYB103*, are able to induce the expression of cellulosic synthesis genes and increase SCW thickening in fibers (Zhong et al., 2008; Hussey et al., 2011). Repression of these three genes, as well as three other MYB TFs, *MYB52*, *MYB54*, and *MYB85* reduced cell wall thickness, supporting the idea that these genes are positive regulators for SCW synthesis (Zhong et al., 2008). Overexpression of *MYB52* and *MYB54* upregulate the expression of *CELLULOSE SYNTHASE 8* (*CesA8*), *IRREGULAR XYLEM 9* (*IRX9*), and 4*-COUMARATE-COA LIGASE* (*4CL*), genes responsible for the synthesis of cellulose, hemicellulose, and lignin, respectively (Zhong et al., 2008). Three MYB TFs, MYB58, MYB63, and MYB85, have been suggested to directly regulate lignin biosynthesis in *Arabidopsis* (Zhong et al., 2008; Zhou et al., 2009). Many regulators in tier 1 are positively regulated by both tier 3 master regulators and tier 2 regulators (Zhong et al., 2008; Ko et al., 2009; Kim et al., 2014; Zhong and Ye, 2014).

### Fine-Tuning of the SCW Regulatory Network

There are some TFs that do not appear to easily fit into SCW-related regulatory networks, which are primarily under feed forward regulation (Taylor-Teeples et al., 2015). *KNOTTED ARABIDOPSIS THALIANA7* (*KNAT7*) is identified as a negative regulator of SCW synthesis. In the *knat7* knockout mutant, irregular xylem vessel formation was observed, but the interfascicular fibers developed thicker SCW (Li et al., 2012). *KANT7* is induced by overexpression of *MYB85* and several NAC master regulators (Zhong et al., 2008). The mechanism of *KANT7* in regulating SCW biosynthesis is still unclear. Another TF *XYLEM NAC DOMAIN 1* (*XND1*) regulates SCW deposition and PCD in xylem, but it is not clear how this gene interacts with other members in regulatory pathways (Zhao et al., 2008). Three MYB TFs, MYB4, MYB7, and MYB32, are negative regulators for the NAC domain master regulators, while the expression of these three MYB genes are positively regulated by the tier2 master regulator MYB46 (Zhong et al., 2008; Ko et al., 2009; Wang and Dixon, 2012; Zhang et al., 2014). These negative regulators may be important to SCW synthesis by providing flexibility under undesirable growth conditions (Jin et al., 2000).

Several regulators have been shown to negatively regulate the NAC domain master regulators. *VND INTERACTING 2* (*VNI2*) directly binds to the VND7 protein, and represses *VND7* expression (Yamaguchi et al., 2010b). Overexpression of *VNI2* leads to failure of xylem vessel development due to inhibition of *VND7*, while mutation of *VNI2* upregulates genes involved in vessel formation (Yamaguchi et al., 2010b). WRKY12 is a negative regulator of the NAC domain regulator NST2 (Wang et al., 2010). In wild-type plants, WRKY12 binds directly to the promoter of *NST2*, resulting in the suppression of SCW biosynthetic genes in pith cells (Wang et al., 2010). Mutation of *WRKY12* de-represses SCW biosynthesis in the pith cells, resulting in a SCW thickening in pith (STP) phenotype (Wang et al., 2010).

Positive regulators have also been identified in regulating NAC domain master regulators. Overexpression of *MYB26* leads to enhanced SCW deposition. Further analysis indicated that MYB26 positively regulate SCW accumulation through NST1 and NST2 (Yang et al., 2007). Recently, a large scale of Yeast one Hybrid (Y1H) screen identified another upstream TF, E2Fc, from the SCW regulatory network. E2Fc can directly bind the promoters of *VND6* and *VND7*, and may function as a positive or negative regulator depends on their relative concentration (Taylor-Teeples et al., 2015).

## Biotechnological Utilization of Discoveries from Model Species

The *Arabidopsis* stem and hypocotyl undergo secondary growth that resembles perennial trees, which makes it a model plant for studying vascular development and wood formation (Chaffey et al., 2002; Nieminen et al., 2004). Indeed, most of the current knowledge of vascular development and xylem differentiation are derived from studies in *Arabidopsis*, or more recently, from a monocot model species *Brachypodium* (Handakumbura and Hazen, 2012). Some of the regulatory genes identified from model plants have conserved functions in biofuel feedstocks (Shen et al., 2009; Zhong et al., 2011). For example, TDIF signaling controls cambial cell divisions in aspen. Precise tissue specific overexpression of the aspen receptor kinase PttPXY and its peptide ligand PttCLE41 exhibited a dramatically increase in tree growth and productivity (Etchells et al., 2015). In another study, significant enhancements in forage biomass and quality were achieved through engineering WRKY TFs in *Zea mays*, *Panicum virgatum*, and *Medicago sativa* (Gallego-Giraldo et al., 2016). The biotechnological utilizations of genes discovered from fundamental research in vascular development and SCW synthesis provide proof of concept for future bioengineering of biofuel feedstocks.

## Concluding Remarks

We discuss the advances in the molecular regulation of vascular development and SCW deposition. Multiple regulatory pathways, such as plant hormones, HD-ZIP III TFs, VND TFs and CLE peptide signaling, have been suggested in regulating procambium development and xylem differentiation. Future studies should focus on the interactions among these pathways. For SCW biosynthesis, NAC domain TFs, MYB domain TFs and many other TFs are members of the gene regulatory network. Even though both positive and negative feedback regulation have been proposed, we know little about the molecular mechanisms of how xylem cells become committed to their identity. In order to fully understand these processes, it is essential to identify novel genes responsible for cambial cell division and xylem differentiation.

## Author Contributions

JY and HW prepared the figures, wrote the manuscript, read and approved the final version.

## Conflict of Interest Statement

The authors declare that the research was conducted in the absence of any commercial or financial relationships that could be construed as a potential conflict of interest.

## References

[B1] Agusti J. Lichtenberger R. Schwarz M. Nehlin L. Greb T. (2011). Characterization of transcriptome remodeling during cambium formation identifies MOL1 and RUL1 as opposing regulators of secondary growth. *PLoS Genet*. 7:e1001312 10.1371/journal.pgen.1001312 PMC3040665 21379334

[B2] Berleth T. Mattsson J. Hardtke C. S. (2000). Vascular continuity and auxin signals. *Trends Plant Sci*. 5 387–393. 10.1016/S1360-1385(00)01725-8 10973094

[B3] Bishopp A. Help H. El-Showk S. Weijers D. Scheres B. Friml J. (2011). A mutually inhibitory interaction between auxin and cytokinin specifies vascular pattern in roots. *Curr. Biol*. 21 917–926. 10.1016/j.cub.2011.04.017 21620702

[B4] Cano-Delgado A. Lee J. Y. Demura T. (2010). Regulatory mechanisms for specification and patterning of plant vascular tissues. *Annu. Rev. Cell Dev. Biol*. 26 605–637. 10.1146/annurev-cellbio-100109-104107 20590454

[B5] Carpita N. C. (2012). Progress in the biological synthesis of the plant cell wall: new ideas for improving biomass for bioenergy. *Curr. Opin. Biotechnol*. 23 330–337. 10.1016/j.copbio.2011.12.003 22209015

[B6] Carroll A. Somerville C. (2009). Cellulosic biofuels. *Annu. Rev. Plant Biol*. 60 165–182. 10.1146/annurev.arplant.043008.092125 19014348

[B7] Chaffey N. Cholewa E. Regan S. Sundberg B. (2002). Secondary xylem development in *Arabidopsis*: a model for wood formation. *Physiol. Plant*. 114 594–600. 10.1034/j.1399-3054.2002.1140413.x 11975734

[B8] Demura T. Ye Z. H. (2010). Regulation of plant biomass production. *Curr. Opin. Plant Biol*. 13 299–304. 10.1016/j.pbi.2010.03.002 20381410

[B9] De Rybel B. Adibi M. Breda A. S. Wendrich J. R. Smit M. E. Novak O. (2014). Plant development. Integration of growth and patterning during vascular tissue formation in *Arabidopsis*. *Science* 345 1255215 10.1126/science.1255215 25104393

[B10] De Rybel B. Moller B. Yoshida S. Grabowicz I. Barbier De Reuille P. Boeren S. (2013). A bHLH complex controls embryonic vascular tissue establishment and indeterminate growth in *Arabidopsis*. *Dev. Cell* 24 426–437. 10.1016/j.devcel.2012.12.013 23415953

[B11] Donner T. J. Sherr I. Scarpella E. (2009). Regulation of preprocambial cell state acquisition by auxin signaling in *Arabidopsis* leaves. *Development* 136 3235–3246. 10.1242/dev.037028 19710171

[B12] Du Q. Avci U. Li S. Gallego-Giraldo L. Pattathil S. Qi L. (2015). Activation of miR165b represses AtHB15 expression and induces pith secondary wall development in *Arabidopsis*. *Plant J*. 83 388–400. 10.1111/tpj.12897 26043238

[B13] Du Q. Wang H. (2015). The role of HD-ZIP III transcription factors and miR165/166 in vascular development and secondary cell wall formation. *Plant Signal. Behav*. 10:e1078955 10.1080/15592324.2015.1078955 PMC4883823 26340415

[B14] Eames A. J. MacDaniels L. H. (1947). *An Introduction to Plant Anatomy*. New York, NY: McGraw-Hill Book Company, Inc.

[B15] Elo A. Immanen J. Nieminen K. Helariutta Y. (2009). Stem cell function during plant vascular development. *Semin. Cell Dev. Biol*. 20 1097–1106. 10.1016/j.semcdb.2009.09.009 19770063

[B16] Emery J. F. Floyd S. K. Alvarez J. Eshed Y. Hawker N. P. Izhaki A. (2003). Radial patterning of *Arabidopsis* shoots by class III HD-ZIP and KANADI genes. *Curr. Biol*. 13 1768–1774. 10.1016/j.cub.2003.09.035 14561401

[B17] Etchells J. P. Mishra L. S. Kumar M. Campbell L. Turner S. R. (2015). Wood formation in trees is increased by manipulating PXY-regulated cell division. *Curr. Biol*. 25 1050–1055. 10.1016/j.cub.2015.02.023 25866390 PMC4406943

[B18] Etchells J. P. Provost C. M. Mishra L. Turner S. R. (2013). WOX4 and WOX14 act downstream of the PXY receptor kinase to regulate plant vascular proliferation independently of any role in vascular organisation. *Development* 140 2224–2234. 10.1242/dev.091314 23578929 PMC3912870

[B19] Etchells J. P. Turner S. R. (2010). The PXY-CLE41 receptor ligand pair defines a multifunctional pathway that controls the rate and orientation of vascular cell division. *Development* 137 767–774. 10.1242/dev.044941 20147378

[B20] Fisher K. Turner S. (2007). PXY, a receptor-like kinase essential for maintaining polarity during plant vascular-tissue development. *Curr. Biol*. 17 1061–1066. 10.1016/j.cub.2007.05.049 17570668

[B21] Friml J. (2003). Auxin transport – shaping the plant. *Curr. Opin. Plant Biol*. 6 7–12. 10.1016/S1369526602000031 12495745

[B22] Funk V. Kositsup B. Zhao C. Beers E. P. (2002). The *Arabidopsis* xylem peptidase XCP1 is a tracheary element vacuolar protein that may be a papain ortholog. *Plant Physiol*. 128 84–94. 10.1104/pp.010514 11788755 PMC148946

[B23] Gallego-Giraldo L. Shadle G. Shen H. Barros-Rios J. Fresquet Corrales S. Wang H. (2016). Combining enhanced biomass density with reduced lignin level for improved forage quality. *Plant Biotechnol. J*. 14 895–904. 10.1111/pbi.12439 26190611 PMC11388942

[B24] Green K. A. Prigge M. J. Katzman R. B. Clark S. E. (2005). CORONA, a member of the class III homeodomain leucine zipper gene family in *Arabidopsis*, regulates stem cell specification and organogenesis. *Plant Cell* 17 691–704. 10.1105/tpc.104.026179 15705957 PMC1069692

[B25] Hamann T. Benkova E. Baurle I. Kientz M. Jurgens G. (2002). The *Arabidopsis* BODENLOS gene encodes an auxin response protein inhibiting MONOPTEROS-mediated embryo patterning. *Genes Dev*. 16 1610–1615. 10.1101/gad.229402 12101120 PMC186366

[B26] Handakumbura P. P. Hazen S. P. (2012). Transcriptional regulation of grass secondary cell wall biosynthesis: playing catch-up with *Arabidopsis thaliana*. *Front. Plant Sci*. 3:74 10.3389/fpls.2012.00074 PMC3355686 22639662

[B27] Hardtke C. S. Berleth T. (1998). The *Arabidopsis* gene MONOPTEROS encodes a transcription factor mediating embryo axis formation and vascular development. *EMBO J*. 17 1405–1411. 10.1093/emboj/17.5.1405 9482737 PMC1170488

[B28] Hirakawa Y. Kondo Y. Fukuda H. (2010). TDIF peptide signaling regulates vascular stem cell proliferation via the WOX4 homeobox gene in *Arabidopsis*. *Plant Cell* 22 2618–2629. 10.1105/tpc.110.076083 20729381 PMC2947162

[B29] Hirakawa Y. Shinohara H. Kondo Y. Inoue A. Nakanomyo I. Ogawa M. (2008). Non-cell-autonomous control of vascular stem cell fate by a CLE peptide/receptor system. *Proc. Natl. Acad. Sci. U.S.A*. 105 15208–15213. 10.1073/pnas.0808444105 18812507 PMC2567516

[B30] Hussey S. G. Mizrachi E. Spokevicius A. V. Bossinger G. Berger D. K. Myburg A. A. (2011). SND2, a NAC transcription factor gene, regulates genes involved in secondary cell wall development in *Arabidopsis* fibres and increases fibre cell area in Eucalyptus. *BMC Plant Biol*. 11:173 10.1186/1471-2229-11-173 PMC3289092 22133261

[B31] Ito Y. Nakanomyo I. Motose H. Iwamoto K. Sawa S. Dohmae N. (2006). Dodeca-CLE peptides as suppressors of plant stem cell differentiation. *Science* 313 842–845. 10.1126/science.1128436 16902140

[B32] Jin H. Cominelli E. Bailey P. Parr A. Mehrtens F. Jones J. (2000). Transcriptional repression by AtMYB4 controls production of UV-protecting sunscreens in *Arabidopsis*. *EMBO J*. 19 6150–6161. 10.1093/emboj/19.22.6150 11080161 PMC305818

[B33] Jouannet V. Brackmann K. Greb T. (2015). (Pro)cambium formation and proliferation: two sides of the same coin? *Curr. Opin. Plant Biol*. 23 54–60. 10.1016/j.pbi.2014.10.010 25449727 PMC4353845

[B34] Kim J. Jung J. H. Reyes J. L. Kim Y. S. Kim S. Y. Chung K. S. (2005). microRNA-directed cleavage of ATHB15 mRNA regulates vascular development in *Arabidopsis* inflorescence stems. *Plant J*. 42 84–94. 10.1111/j.1365-313X.2005.02354.x 15773855 PMC1382282

[B35] Kim W. C. Kim J. Y. Ko J. H. Kang H. Han K. H. (2014). Identification of direct targets of transcription factor MYB46 provides insights into the transcriptional regulation of secondary wall biosynthesis. *Plant Mol. Biol*. 85 589–599. 10.1007/s11103-014-0205-x 24879533

[B36] Ko J. H. Kim W. C. Han K. H. (2009). Ectopic expression of MYB46 identifies transcriptional regulatory genes involved in secondary wall biosynthesis in *Arabidopsis*. *Plant J*. 60 649–665. 10.1111/j.1365-313X.2009.03989.x 19674407

[B37] Kondo Y. Fujita T. Sugiyama M. Fukuda H. (2015). A novel system for xylem cell differentiation in *Arabidopsis thaliana*. *Mol. Plant* 8 612–621. 10.1016/j.molp.2014.10.008 25624147

[B38] Kondo Y. Ito T. Nakagami H. Hirakawa Y. Saito M. Tamaki T. (2014a). Plant GSK3 proteins regulate xylem cell differentiation downstream of TDIF-TDR signalling. *Nat. Commun*. 5:3504 10.1038/ncomms4504 24662460

[B39] Kondo Y. Tamaki T. Fukuda H. (2014b). Regulation of xylem cell fate. *Front. Plant Sci*. 5:315 10.3389/fpls.2014.00315 PMC4076795 25071798

[B40] Kubo M. Udagawa M. Nishikubo N. Horiguchi G. Yamaguchi M. Ito J. (2005). Transcription switches for protoxylem and metaxylem vessel formation. *Genes Dev*. 19 1855–1860. 10.1101/gad.1331305 16103214 PMC1186185

[B41] Li E. Bhargava A. Qiang W. Friedmann M. C. Forneris N. Savidge R. A. (2012). The Class II KNOX gene KNAT7 negatively regulates secondary wall formation in *Arabidopsis* and is functionally conserved in *Populus*. *New Phytol*. 194 102–115. 10.1111/j.1469-8137.2011.04016.x 22236040

[B42] Li J. Nam K. H. (2002). Regulation of brassinosteroid signaling by a GSK3/SHAGGY-like kinase. *Science* 295 1299–1301. 11847343 10.1126/science.1065769

[B43] Mazur E. Kurczynska E. U. Friml J. (2014). Cellular events during interfascicular cambium ontogenesis in inflorescence stems of *Arabidopsis*. *Protoplasma* 251 1125–1139. 10.1007/s00709-014-0620-5 24526327

[B44] McConnell J. R. Emery J. Eshed Y. Bao N. Bowman J. Barton M. K. (2001). Role of PHABULOSA and PHAVOLUTA in determining radial patterning in shoots. *Nature* 411 709–713. 10.1038/35079635 11395776

[B45] Mitsuda N. Iwase A. Yamamoto H. Yoshida M. Seki M. Shinozaki K. (2007). NAC transcription factors, NST1 and NST3, are key regulators of the formation of secondary walls in woody tissues of *Arabidopsis*. *Plant Cell* 19 270–280. 10.1105/tpc.106.047043 17237351 PMC1820955

[B46] Mitsuda N. Seki M. Shinozaki K. Ohme-Takagi M. (2005). The NAC transcription factors NST1 and NST2 of *Arabidopsis* regulate secondary wall thickenings and are required for anther dehiscence. *Plant Cell* 17 2993–3006. 10.1105/tpc.105.036004 16214898 PMC1276025

[B47] Miyashima S. Sebastian J. Lee J. Y. Helariutta Y. (2013). Stem cell function during plant vascular development. *EMBO J*. 32 178–193. 10.1038/emboj.2012.301 23169537 PMC3553377

[B48] Nieminen K. M. Kauppinen L. Helariutta Y. (2004). A weed for wood? *Arabidopsis* as a genetic model for xylem development. *Plant Physiol*. 135 653–659. 10.1104/pp.104.040212 15208411 PMC514101

[B49] Ohashi-Ito K. Fukuda H. (2014). Xylem. *Curr. Biol*. 24:R1149 10.1016/j.cub.2014.10.010 25514001

[B50] Ohashi-Ito K. Oda Y. Fukuda H. (2010). *Arabidopsis* VASCULAR-RELATED NAC-DOMAIN6 directly regulates the genes that govern programmed cell death and secondary wall formation during xylem differentiation. *Plant Cell* 22 3461–3473. 10.1105/tpc.110.075036 20952636 PMC2990123

[B51] Ohashi-Ito K. Oguchi M. Kojima M. Sakakibara H. Fukuda H. (2013). Auxin-associated initiation of vascular cell differentiation by LONESOME HIGHWAY. *Development* 140 765–769. 10.1242/dev.087924 23362345

[B52] Ohashi-Ito K. Saegusa M. Iwamoto K. Oda Y. Katayama H. Kojima M. (2014). A bHLH complex activates vascular cell division via cytokinin action in root apical meristem. *Curr. Biol*. 24 2053–2058. 10.1016/j.cub.2014.07.050 25131670

[B53] Pauly M. Keegstra K. (2010). Plant cell wall polymers as precursors for biofuels. *Curr. Opin. Plant Biol*. 13 305–312. 10.1016/j.pbi.2009.12.009 20097119

[B54] Prigge M. J. Otsuga D. Alonso J. M. Ecker J. R. Drews G. N. Clark S. E. (2005). Class III homeodomain-leucine zipper gene family members have overlapping, antagonistic, and distinct roles in *Arabidopsis* development. *Plant Cell* 17 61–76. 10.1105/tpc.104.026161 15598805 PMC544490

[B55] Ruzicka K. Ursache R. Hejatko J. Helariutta Y. (2015). Xylem development – from the cradle to the grave. *New Phytol*. 207 519–535. 10.1111/nph.13383 25809158

[B56] Schlereth A. Moller B. Liu W. Kientz M. Flipse J. Rademacher E. H. (2010). MONOPTEROS controls embryonic root initiation by regulating a mobile transcription factor. *Nature* 464 913–916. 10.1038/nature08836 20220754

[B57] Schuetz M. Smith R. Ellis B. (2013). Xylem tissue specification, patterning, and differentiation mechanisms. *J. Exp. Bot*. 64 11–31. 10.1093/jxb/ers287 23162114

[B58] Shen H. Yin Y. B. Chen F. Xu Y. Dixon R. A. (2009). A bioinformatic analysis of NAC genes for plant cell wall development in relation to lignocellulosic bioenergy production. *Bioenergy Res*. 2 217–232. 10.1007/s12155-009-9047-9

[B59] Somerville C. (2007). Biofuels. *Curr. Biol*. 17 R115–R119. 10.1016/j.cub.2007.01.010 17307040

[B60] Taylor-Teeples M. Lin L. De Lucas M. Turco G. Toal T. W. Gaudinier A. (2015). An *Arabidopsis* gene regulatory network for secondary cell wall synthesis. *Nature* 517 571–575. 10.1038/nature14099 25533953 PMC4333722

[B61] Ursache R. Miyashima S. Chen Q. Vaten A. Nakajima K. Carlsbecker A. (2014). Tryptophan-dependent auxin biosynthesis is required for HD-ZIP III-mediated xylem patterning. *Development* 141 1250–1259. 10.1242/dev.103473 24595288 PMC7055496

[B62] Wang H. Avci U. Nakashima J. Hahn M. G. Chen F. Dixon R. A. (2010). Mutation of WRKY transcription factors initiates pith secondary wall formation and increases stem biomass in dicotyledonous plants. *Proc. Natl. Acad. Sci. U.S.A*. 107 22338–22343. 10.1073/pnas.1016436107 21135241 PMC3009815

[B63] Wang H. Zhao Q. Chen F. Wang M. Dixon R. A. (2011). NAC domain function and transcriptional control of a secondary cell wall master switch. *Plant J*. 68 1104–1114. 10.1111/j.1365-313X.2011.04764.x 21883551

[B64] Wang H. Z. Dixon R. A. (2012). On-off switches for secondary cell wall biosynthesis. *Mol. Plant* 5 297–303. 10.1093/mp/ssr098 22138968

[B65] Wang J. Kucukoglu M. Zhang L. Chen P. Decker D. Nilsson O. (2013). The *Arabidopsis* LRR-RLK, PXC1, is a regulator of secondary wall formation correlated with the TDIF-PXY/TDR-WOX4 signaling pathway. *BMC Plant Biol*. 13:94 10.1186/1471-2229-13-94 PMC3716795 23815750

[B66] Wenzel C. L. Schuetz M. Yu Q. Mattsson J. (2007). Dynamics of MONOPTEROS and PIN-FORMED1 expression during leaf vein pattern formation in *Arabidopsis thaliana*. *Plant J*. 49 387–398. 10.1111/j.1365-313X.2006.02977.x 17217464

[B67] Williams L. Grigg S. P. Xie M. Christensen S. Fletcher J. C. (2005). Regulation of *Arabidopsis* shoot apical meristem and lateral organ formation by microRNA miR166g and its AtHD-ZIP target genes. *Development* 132 3657–3668. 10.1242/dev.01942 16033795

[B68] Yamaguchi M. Goue N. Igarashi H. Ohtani M. Nakano Y. Mortimer J. C. (2010a). VASCULAR-RELATED NAC-DOMAIN6 and VASCULAR-RELATED NAC-DOMAIN7 effectively Induce transdifferentiation into xylem vessel elements under control of an induction system. *Plant Physiol*. 153 906–914. 10.1104/pp.110.154013 20488898 PMC2899931

[B69] Yamaguchi M. Mitsuda N. Ohtani M. Ohme-Takagi M. Kato K. Demura T. (2011). VASCULAR-RELATED NAC-DOMAIN7 directly regulates the expression of a broad range of genes for xylem vessel formation. *Plant J*. 66 579–590. 10.1111/j.1365-313X.2011.04514.x 21284754

[B70] Yamaguchi M. Ohtani M. Mitsuda N. Kubo M. Ohme-Takagi M. Fukuda H. (2010b). VND-INTERACTING2, a NAC domain transcription factor, negatively regulates xylem vessel formation in *Arabidopsis*. *Plant Cell* 22 1249–1263. 10.1105/tpc.108.064048 20388856 PMC2879754

[B71] Yang C. Xu Z. Song J. Conner K. Vizcay Barrena G. Wilson Z. A. (2007). *Arabidopsis* MYB26/MALE STERILE35 regulates secondary thickening in the endothecium and is essential for anther dehiscence. *Plant Cell* 19 534–548. 10.1105/tpc.106.046391 17329564 PMC1867336

[B72] Yin Y. Wang Z. Y. Mora-Garcia S. Li J. Yoshida S. Asami T. (2002). BES1 accumulates in the nucleus in response to brassinosteroids to regulate gene expression and promote stem elongation. *Cell* 109 181–191. 10.1016/S0092-8674(02)00721-3 12007405

[B73] Zhang J. Nieminen K. Serra J. A. Helariutta Y. (2014). The formation of wood and its control. *Curr. Opin. Plant Biol*. 17 56–63. 10.1016/j.pbi.2013.11.003 24507495

[B74] Zhao C. Avci U. Grant E. H. Haigler C. H. Beers E. P. (2008). XND1, a member of the NAC domain family in *Arabidopsis thaliana*, negatively regulates lignocellulose synthesis and programmed cell death in xylem. *Plant J*. 53 425–436. 10.1111/j.1365-313X.2007.03350.x 18069942

[B75] Zhong R. Demura T. Ye Z. H. (2006). SND1, a NAC domain transcription factor, is a key regulator of secondary wall synthesis in fibers of *Arabidopsis*. *Plant Cell* 18 3158–3170. 10.1105/tpc.106.047399 17114348 PMC1693950

[B76] Zhong R. Lee C. Mccarthy R. L. Reeves C. K. Jones E. G. Ye Z. H. (2011). Transcriptional activation of secondary wall biosynthesis by rice and maize NAC and MYB transcription factors. *Plant Cell Physiol*. 52 1856–1871. 10.1093/pcp/pcr123 21908441

[B77] Zhong R. Lee C. Zhou J. Mccarthy R. L. Ye Z. H. (2008). A battery of transcription factors involved in the regulation of secondary cell wall biosynthesis in *Arabidopsis*. *Plant Cell* 20 2763–2782. 10.1105/tpc.108.061325 18952777 PMC2590737

[B78] Zhong R. Richardson E. A. Ye Z. H. (2007a). The MYB46 transcription factor is a direct target of SND1 and regulates secondary wall biosynthesis in *Arabidopsis*. *Plant Cell* 19 2776–2792. 10.1105/tpc.107.053678 17890373 PMC2048704

[B79] Zhong R. Richardson E. A. Ye Z. H. (2007b). Two NAC domain transcription factors, SND1 and NST1, function redundantly in regulation of secondary wall synthesis in fibers of *Arabidopsis*. *Planta* 225 1603–1611. 10.1007/s00425-007-0498-y 17333250

[B80] Zhong R. Ye Z. H. (2012). MYB46 and MYB83 bind to the SMRE sites and directly activate a suite of transcription factors and secondary wall biosynthetic genes. *Plant Cell Physiol*. 53 368–380. 10.1093/pcp/pcr185 22197883

[B81] Zhong R. Ye Z. H. (2014). Complexity of the transcriptional network controlling secondary wall biosynthesis. *Plant Sci*. 229 193–207. 10.1016/j.plantsci.2014.09.009 25443846

[B82] Zhong R. Ye Z. H. (2015). Secondary cell walls: biosynthesis, patterned deposition and transcriptional regulation. *Plant Cell Physiol*. 56 195–214. 10.1093/pcp/pcu140 25294860

[B83] Zhou J. Lee C. Zhong R. Ye Z. H. (2009). MYB58 and MYB63 are transcriptional activators of the lignin biosynthetic pathway during secondary cell wall formation in *Arabidopsis*. *Plant Cell* 21 248–266. 10.1105/tpc.108.063321 19122102 PMC2648072

[B84] Zhou J. Zhong R. Ye Z. H. (2014). *Arabidopsis* NAC domain proteins, VND1 to VND5, are transcriptional regulators of secondary wall biosynthesis in vessels. *PLoS ONE* 9:e105726 10.1371/journal.pone.0105726 PMC4141820 25148240

[B85] Zhou Y. Liu X. Engstrom E. M. Nimchuk Z. L. Pruneda-Paz J. L. Tarr P. T. (2015). Control of plant stem cell function by conserved interacting transcriptional regulators. *Nature* 517 377–380. 10.1038/nature13853 25363783 PMC4297503

